# An examination of the sexual functions of patients who underwent a gynecologic cancer operation and received brachytherapy

**DOI:** 10.12669/pjms.341.14241

**Published:** 2018

**Authors:** Ozlem Guner, Sureyya Gumussoy, Nigar Celik, Aynur Saruhan, Oya Kavlak

**Affiliations:** 1 Dr. Ozlem Guner, Department of Women's Health and Gynecology, Faculty of Nursing, Ege University, Izmir, Turkey; 2 Dr. Sureyya Gumussoy, Department of Women's Health and Gynecology, Faculty of Nursing, Ege University, Izmir, Turkey; 3 Dr. Nigar Celik, Department of Women's Health and Gynecology, Faculty of Nursing, Ege University, Izmir, Turkey; 4 Aynur Saruhan, Assistant Professor, Department of Women's Health and Gynecology, Faculty of Nursing, Ege University, Izmir, Turkey; 5 Prof. Oya Kavlak, Department of Women's Health and Gynecology, Faculty of Nursing, Ege University, Izmir, Turkey

**Keywords:** Brachytherapy, Gynecologic Cancers, Sexual Function

## Abstract

**Objective:**

This study was planned as a descriptive study for the purpose of examining the sexual functions of patients' who underwent a gynecological operation and received brachytherapy.

**Methods:**

The study was conducted with 118 women who attended the Radiation Oncology Unit at Ege University Medical Faculty Hospital in Izmir Province for Gynecological Oncology follow-up, who participated voluntarily and were assigned using the random sampling method. The participants were married, sexually active, had a diagnosis of gynecologic cancer, underwent an operation and received brachytherapy for four months after the operation. The Individual Identification Form and Female Sexual Function Index (FSFI) were used as the data collection tools.

**Results:**

The average age of women who participated in study was 50.90±7.98 and 41.5% of them had completed primary school. About 60% of the participants had cervical cancer and 69.5% had a total abdominal hysterectomy with bilateral salpingo-oopherectomy. The FSFI average score was determined to be 15.77±8.71. It was found that 97.5% of the participants received fewer than 30 points from the scale and these participants thus experienced sexual dysfunction.

**Conclusions:**

According to the findings obtained from the study, almost all thewomen that had an operation as a result of adiagnosis of gynecologic cancer and received brachytherapy experienced sexual dysfunction.

## INTRODUCTION

Cancer has always been one of the most important health problems because of its frequent incidence and high lethality.^1^ About 14.1 million new cases of cancer and 8.2 million deaths resulting from cancer were detected in 2012.^2^ Cancer is thus a health problem which is high in prevalence and mortality and it also has asignificant place among diseases affecting women. Gynecologic cancers are the most important cause of morbidity and mortality in women after breast cancer. Gynecologic cancer is the fourth most frequent type of cancer in the World.^3^,^4^

The greatest adverse effect of gynecologic cancer treatments occurs to sexual functioning.^5^ Brachytherapy and radiotherapy are applied in gynecologic cancers. Radiation is directly given to the tumor or to the cavity close to the tumor in brachytherapy, so a small part of the body is primarily affected in this situation.^4^ Pelvic radiation therapy causes ovarian failure, although the severity of this varies depending upon the radiotherapy dose and the patient's age. Ovarian failure based on a decrease in estradiol and progesteronecauses symptoms of early menopause, loss of libido and postcoital hemorrhage.^6^ In addition, radiotherapy temporarily damages the epithelium of the vagina, vascular structures and connective tissue fibroblasts^7^ and causes a number of changes in the vaginal wall.^6^

These changes intensify over a period of weeks and transform into scar tissue, causing vaginal obstruction and a decrease in elasticity. This leads to pain, bleeding and other sexual problems during sexual intercourse.^4^ Sexual dysfunction may harm the relationship between the patient and her partner and add a further problem to those that the individual suffering from cancer already experiences. In addition to the care received from medical personnel, a suitable counseling/advisoryservice can thus play a key role to increase the patient's quality of life.^8^

This study was therefore planned to assess the sexual functioning of patients' who had undergone a gynecologic operation and received brachy therapy, and at the same time to examine the factors affecting their sexual functioning.

## METHODS

The study, which was designed using the random sampling method, was conducted with 118 women who participated voluntarily, out of a total of 800 who attended the Radiation Oncology Unit, Ege University Medical Faculty Hospital, Izmir Province between April 2013 and April 2014 for the purpose of Gynecologic Oncology follow-up. The women were married, sexually active, had a diagnosis of gynecologic cancer, had an operation and had received brachy therapy for at least four months after the operation. The permission of the patients was obtained after they had been informed about the purpose of the study. The Ethics Committee of the Ege University of Nursing Faculty approved the study protocol (Ethics committee approval number; 2013-28). This study was planned as definer.

### Data Collection

The Individual Identification Form and the Female Sexual Function Index (FSFI), which determine socio-demographic characteristics and other variables were used as the means of data collection.

### Individual Identification Form

The Individual Identification Form consists of a total 36 questions formulatedin accordance with the related literature, including questions about the socio-demographic characteristics and medical diagnoses of the women, the operations they have undergone and the treatments received, their sex life before the operation, and changes in their sex life after treatment.^7^,^9^

### Female Sexual Function Index (FSFI)

The Female Sexual Function Index, whose validity and reliability study was carried out by Yilmaz and Eryilmaz in 2004^10^, consists of 9 questions. Each question examines the status of the woman's sexual functionin the last 4 weeks. The highest score that can be obtained from the scale is 49 and the cut-off point is 30. Sexual dysfunction is said to arise below 30 points. There are three sub-dimensions in the scale; these are “sexual satisfaction” (items 6, 7, 8), “the frequency of sexual intercourse” (items 3, 4, 5, 9) and “discomfort in sexual intercourse” (items 1, 2). According to the results of the validity study the FSFI enables the examination of desire, arousal, orgasm and areas of pain in sexual intercourse, and the Cronbach's Alpha value, which is the internal consistency coefficient of the scale, was found to be 0.82. In our study, the Cronbach's Alpha value was determined to be 0.97.

### Analysis

The IBM SPSS (Statistical Package for Social Sciences) for Windows 20 was used to statistically evaluate the data obtained from the study. Data obtained from the study were evaluated using number, percentage, average, standard deviation, median, Mann-Whitney U, Kruskall Wallis and the independent t-test. Correlation analysis was performed to determine the relation of multiple variables with each other. The results were evaluated at a 95% confidence interval and p<0.05 significance level.

## RESULTS

One hundred eighteen women were included in the study. All of the participants were married, their ages ranged between 36 and 68, and their average age was found to be 50.9±7.98. 41.5% (n=49) of the participants were found to have completed primary school. The average number of pregnancies of the participants was found to be 3.61±1.69, the average number of births 2.89±1.16 and the average number of living children 2.7±1.1. 68.6% (n=81) of the patients were found to have gone through the menopause after the surgery. About 60.2% (n=71) of the participants were found to have cervical cancer, 39.8% (n=47) of them had endometrial carcinoma, and 42.4% of these were in the second phase of the FIGO cancer classification. 69.5% (n=82) of the participants had a total abdominal hysterectomy with bilateral salpingo-oopherectomy. 24.8% (n=29) of them received only brachytherapy, 40.2% of them had brachytherapy+chemotherapy, 20.5% (n=24) of them had brachytherapy+radiotherapy and 14.5% (n=17) of them had combined modality therapy (brachytherapy+chemotherapy+radiotherapy) after the operation. About 52.5% (n=62) of the patients said that they were scared when they learned that they would have an operation and 74.1% (n=83) answered the question “What made you feel like this?” with “The name of the disease”. It was determined that 54.2% (n=64) of the patients did not have any questions about the changes that could occur in their post-operative sex lives and this is because 59.5% (n=44) of them thought that their health problems were more important than their sex lives. About 89.8% (n=106) of the participants experienced changes in their sex lives after the treatment, 43.4% (n=46) of them stated that they did not feel as much sexual desire as before and 36.8% (n=39) of them indicated that they had a lot of pain during intercourse.

The average score that the patients received from the FSFI was 15.77±8.71. The average scores from the subgroups in relation to FSFI were: “sexual satisfaction”, 4.87±3.38; “the frequency of sexual intercourse”, 6.64±3.42; and “discomfort during sexual intercourse”, 4.25± 2.2. The relationships between some characteristics of the patients and the Female Sexual Function Index (FSFI) and it`s subdimensions are shown in Table-I.

**Table-I:** IFSF sexual function and subscore status according to some characteristics of patients.

Feature	N	%	Sexual satisfaction	Sexual intercourse frequency libido	Sexual discomfort	IFSF (toplampuan)
*Education*						
Literate	9	7.6	X^2^=25.132 p=0.001	X^2^=18.620 p=0.001	X^2^=21.985 p=0.001	X^2^=24.163 p=0.001
Primary school graduate	49	41.5				
Middle School	15	12.7				
High school	27	22.9				
Faculty	18	15.3				
*Participants' working status*						
Working	27	22.9	U=2.723	U=2.675	U=2.122	U=41.696
Not working	91	77.1	p=0.01	p=0.011	p=0.039	p=0.011
*Participants' income situation*						
Less than $ 500	6	5.1	X^2^=7.871 p=0.02	X^2^=5.201 p=0.007	X^2^=7.695 p=0.02	X^2^=7.434 p=0.02
Between $ 500-1000	28	23.7				
Over $ 1000	84	71.2				
Participants smoking status						
Smoking	24	20.3	X^2^=9.934 p=0.007	X^2^=9.081 p=0.011	X^2^=4.807 p=0.09	X^2^=9.145 p=0.01
Non Smoking	70	59.3				
He does not drink after his illness	24	20.4				
Participants Drinking Alcohol						
I've never used	91	77.1	t=-3.267	t=-3.449	t=-2.994	t=-3.390
I rarely use	27	22.9	p=0.001	p=0.001	p=0.003	p=0.001
*Menopause*						
Before Surgery	37	30.8	t=-1.856	t=-1.682	t=-2.523	t=-2.018
After Surgery	81	67.5	p=0.066	p=0.095	p=0.013	p=0.046
*Treatment*						
brachytherapy	29	24.8	X^2^=12.832 p=0.005	X^2^=15.970 p=0.001	X^2^=16.703 p=0.001	X^2^=14.153 p=0.003
brachytherapy + chemotherapy	47	40.2				
brachytherapy+radiotherapy	24	20.5				
brachytherapy+chemotherapy+ radiotherapy	18	14.5				

Total	118	100.0				

The difference between the treatment type and the sexual function scores was determined to stem from the fact that the sexual function scores of patients who received only brachytherapy after surgery were higher than those who received “radiotherapy + brachytherapy” (p = 0.001).

## DISCUSSION

The World Health Organization reports that the prevalence of cancer increases with increasing age.^2^ According to data in 2006, “uterine corpus cancer” is the fourth most common cancer in Turkey among women with a prevalence of 8.4 per 100,000, “ovarian cancer” is the seventh most common with a prevalence of 5.9 per 100,000, and “cervical cancer” is the ninth most common with a prevalence of 4.8 per 100,000.^11^ In the present study, it was determined that 60.2% of the patients had cervical cancer and 39.8% of them had endometrial carcinoma. It is thought that the reason why there were more patients who had cervical cancer in our study can be explained as a result of the increase in the application of brachytherapy in cervical cancer, which has ahigh rate of early diagnosis.

The average score that the patients obtained from the FSFI was 15.77±8.71. In scoring the scale, 30 points or fewer indicates sexual dysfunction. It was determined that 97.5% of the participants had fewer than 30 points, so they had sexual dysfunction. Almost all of the women who participated in the study stated that they had experienced a negative change in their sex lives after treatment. Similarly, when the patients' results before and after a diagnosis of cancer were compared in a study of gynecologic cancer patients by Zeng et al. (2012), it was stated that 19.9% experienced “a lot of changes” in their sexual desire, 10.3% in their vaginal dryness, 9.6% in their vaginal width, 7.7% in pain during the sexual intercourse and 19.9 in their frequency of sexual intercourse.^12^

Radiotherapy, which plays an important role in the treatment of gynecologic cancers, can be applied only as brachytherapy or as external radiotheraphy in combination with brachytherapy.^13^ The application of radiotherapy and brachytherapy in particular, besides vaginal dryness and numbness, has also been reported to cause a decrease in lubrication, sexual interest, satisfaction after sex, and also dyspareunia and problems with orgasm.^14^,^15^ Radiotherapy that is applied in early stage cervical cancer has been stated to cause more sexual problems than surgical treatment.^16^ In another study conducted with patients with cervical cancer, brachytherapy was reported to cause less vaginal lubrication, decrease in genital swelling and vaginal elasticity in comparison with radiotherapy and surgical treatment.^17^ In our study, similarly to the literature, it was found that the patients who received only postoperative brachytherapy had higher sexual function scores than the ones who had postoperative “brachytherapy+radiotherapy”(p=0,001). Similarly, in a study conducted in patients who had early phase endometrial carcinoma, there was no significant difference in terms of their sex lives between the patients who only had a “surgery” and those who received “surgery+brachytheraphy”.^18^ Differently from these results, in other studies conducted brachytheraphy has been reported to increase dysfunctions after each practice and in cases of dose escalation.^19^,^20^

Although it is known that gynecologic cancer patients' sexual functions are affected by the side effects of the treatment they have received and structural-physiological changes in genital organs, health care professionals generally fail to recognize these patients' sexual needs.^8^ Diagnosis of gynecologic cancer and surgical procedures such as radical hysterectomy and vulvectomy, as well as treatments such as brachytherapy, radiotherapy and chemotherapy, cause significant health problems affecting a woman's body image, self-respect and sex life with her partner.^21^,^22^ It was stated in one study that a women's sex life is affected by 25% in breast cancers and 80% in gynecologic cancers.^23^ In this study, it was determined that two-thirds of the patients with questions related to their sex lives were unable to ask anybody these questions. When the reasons for this were examined, more than half of them thought that their health problems were more important, the remaining participants thought these topics could not be discussed and also stated they did not know who to ask. Similarly to this finding obtained in our study, Flynn et al. (2012) determined that 69% of the patients never asked the nurse or doctor about the problems with their sex lives.^24^ In the same study, the reasons for this were that 21% of the patients thought their sex life was not too bad, 2% of them observed that the nurse or the doctor was too busy, 9% of them were ashamed, 3% observed that the nurse or the doctor was of the opposite sex, although fewer than 1% thought the nurse or the doctor was too young for them to mention their problems.

## CONCLUSION

As a result of this study, it was found that almost all of the patients who underwent a gynecologic cancer operation and received brachytherapy experienced sexual dysfunctions. Based on these results, it is thought that women who undergo an operation as a result of a diagnosis of gynecologic cancer and who receive brachytheraphy should be routinely evaluated in terms of sexual dysfunctions, and that in addition to the treatment for cancer, information and counseling should be given to the woman and to her partner about how to maintain their sex lives.

### Authors' Contribution

**OG** conceived, designed and did statistical analysis & editing of manuscript.

**OG, SG and NG** did data collection and manuscript writing.

**OG, SG, OK, and AS** did review and final approval of manuscript.
